# Clinical outcomes between elderly ESKD patients under peritoneal dialysis and hemodialysis: a national cohort study

**DOI:** 10.1038/s41598-023-43476-1

**Published:** 2023-09-27

**Authors:** Yu-Kai Peng, Tzong-Shyuan Tai, Chao-Yi Wu, Chung-Ying Tsai, Cheng-Chia Lee, Jia-Jin Chen, Ching-Chung Hsiao, Yung-Chang Chen, Huang-Yu Yang, Chieh-Li Yen

**Affiliations:** 1 Division of Nephrology, Nephrology Department, Kidney Research Center, Linkou Medical Center, Kidney Research Institute, Chang Gung Memorial Hospital, No.5, Fuxing Street, Guishan District, Taoyuan City, 33305 Taiwan; 2 College of Medicine, Chang Gung University, Taoyuan, Taiwan; 3 Division of Rheumatology, Allergy and Immunology, Department of Internal Medicine, Linkou Chang Gung Memorial Hospital, Taoyuan City, Taiwan; 4 Division of Allergy, Asthma, and Rheumatology, Department of Pediatrics, Chang Gung Memorial Hospital, Taoyuan, Taiwan; 5 Department of Health Policy and Management, Johns Hopkins University Bloomberg School of Public Health, Baltimore, MD USA

**Keywords:** Cardiology, Nephrology

## Abstract

With ageing populations, new elderly end-stage kidney disease (ESKD) cases rise. Unlike younger patients, elderly ESKD patients are less likely to undergo kidney transplant, and therefore the decision of receiving peritoneal dialysis (PD) and hemodialysis (HD) is more crucial. A total of 36,852 patients, aged more than 65, who were newly diagnosed with ESKD and initiated renal replacement therapy between 2013 and 2019 were identified. These patients were categorized into two groups: the PD group and the HD group according to their long-term renal replacement treatment. After propensity score matching, the PD group (*n* = 1628) displayed a lower incidence of major adverse cardiac and cerebrovascular events (MACCE) (10.09% vs. 13.03%, hazard ratio (HR): 0.74, 95% confidence interval (CI): 0.66–0.83), malignancy (1.23% vs. 2.14%, HR: 0.55, 95% CI: 0.40–0.76), and MACCE-associated mortality (1.35% vs. 2.25%, HR: 0.62, 95% CI: 0.46–0.84) compared to the HD group (*n* = 6512). However, the PD group demonstrated a higher rate of infection (34.09% vs. 24.14%, HR: 1.28, 95% CI: 1.20–1.37). The risks of all-cause mortality and infection-associated mortality were not different. This study may provide valuable clinical information to assist elderly ESKD patients to choose HD or PD as their renal replacement therapy.

For patients who are newly diagnosed with end-stage kidney disease (ESKD) and require renal replacement therapy, kidney transplantation has been proven to be the most effective treatment, regardless of whether it is from a living donor or a cadaveric donor^1–3^. However, suitable donors are not always available, and most ESKD patients still need a waiting time before successful transplantation. According to a report from Taiwan in 2019, the average waiting time for a kidney transplant was 4.7 years^4^. Thus, to decide to receive hemodialysis or peritoneal dialysis (PD), which are the two major renal replacement therapy, is inevitable for most patients with new-onset ESKD. Hemodialysis involves the use of an artificial semi-permeable membrane, known as a dialyzer, to remove uremic toxins from the patient's blood. This process typically lasts for around 4 h and requires complex equipment such as a dialysis machine to pump blood into the dialyzer and a water filter system to prevent bacteria or toxins from entering the patient’s bloodstream. Skilled professionals are also needed to perform tasks like arteriovenous fistula (AVF) puncture^5^. Except for certain limited areas, most hemodialysis patients are required to receive treatment at specific facilities, such as dialysis centers or hospitals^6^. On the other hand, PD, which removed uremia toxin through patients’ own peritoneum via infusion of dialysate into abdominal cavity and release it after several hours of dwelling time. The requirement of technic and equipment of PD is relatively simple compared to hemodialysis and thus could be performed by patient self or families. Since PD is a home-based treatment, compared to Hemodialysis, it may have less impact on patients' original lifestyle and may improve health-related quality of life^7,8^.

Aging is a crucial and continuous global healthcare issue in these decades, for example, in Taiwan, people aged more than 65 will account for more than 20% of the total population by 2025^9,10^. Similarly, the number of elderly patients with newly diagnosed end-stage kidney disease (ESKD) has been rapidly increasing, and this trend shows no sign of slowing down in the coming years^4^. The percentage of ESKD patients aged more than 65 has exceeded 50% of whole ESKD population in Taiwan by 2017^4^. Due to the risks associated with surgery and anesthesia, as well as factors like performance and cognitive status, polypharmacy (including drug-drug interactions with immunosuppressants), and shorter life expectancy, elderly ESKD patients have a lower rate of receiving kidney transplants^11,12^. As a result, the decision to receive either hemodialysis or PD, both of which may be lifelong treatments, becomes even more crucial for elderly ESKD patients compared to younger patients. Along with patients aging, the progressively difficult mobility^13^, cognitive decline, frailty, and susceptible to infectious disease make PD, a home-based treatment, which may help the elderly ESKD patients to remain in their community and maintain their original daily life, an attractive option. However, to make an informed decision, elderly ESKD patients need more objective information about crucial outcomes, such as mortality rates, risks of cardiovascular events, infection diseases, and malignancy, which are of utmost importance for this age group. Currently, there is only limited research available that evaluates these essential outcomes between hemodialysis and PD among elderly ESKD patients, and the results are inconsistent. Therefore, a large-scale comprehensive study is warranted for better understanding the following outcomes after receiving maintenance hemodialysis or PD among elderly patients.

Until 2019, according to data from National Healthcare Insurance Research database (NHIRD), approximately 86,840 patients underwent dialysis in Taiwan, of which about 6,901 patients received peritoneal dialysis^4^. Moreover, NHIRD can provide comprehensive and detailed clinical information about these patients. By utilizing these abundant data, this study is aimed to evaluate all-cause mortality, CV outcomes, malignancy risks, and frequency of hospitalizations between PD and HD among ESKD patients aged more than 65.

## Materials and methods

### Data source

The patient data of this study were obtained from NHIRD. Taiwan’s National Health Insurance (NHI) program, which is a single-payer, mandatory enrollment system, have launched since March 1, 1995. In this system, all medical costs are covered by a single public entity, and all medical institutions in Taiwan are required to join in^14^ As a result, this program covers nearly 100% of Taiwan's population. Furthermore, the NHIRD provides academic units and scholars with access for research in medical and public health-related fields. After application, the NHIRD provides detailed information for researchers, including outpatient visits, hospitalizations, disease diagnoses, surgeries, and the drug use. Regarding diagnoses, the International Classification of Diseases, Ninth Edition, Clinical Modification (ICD-9-CM) was used before 2016 and ICD-10 has been adopted thereafter. Moreover, in Taiwan, the patients with new-onset ESKD would obtain certifications as having catastrophic illness and then the copayment of dialysis would be covered by NHI. All applications for this catastrophic illness certification are comprehensively reviewed by experts, thereby ensuring a high degree of diagnostic accuracy. As a result, the data from the catastrophic illness file has been extensively utilized for case verification in various related studies. The NHIRD has replaced the names or identifications of patients, healthcare providers, and medical institutions with anonymous numbers to protect patient privacy^15^. Thus, because this was a database study by using the NHIRD, the requirement for written informed consent was waived. This study has been performed in accordance with the Declaration of Helsinki and was approved by the Institutional Review Board of the Chang Gung Medical Foundation (IRB number: 201900840B0).

### Study design

**Figure 1:**
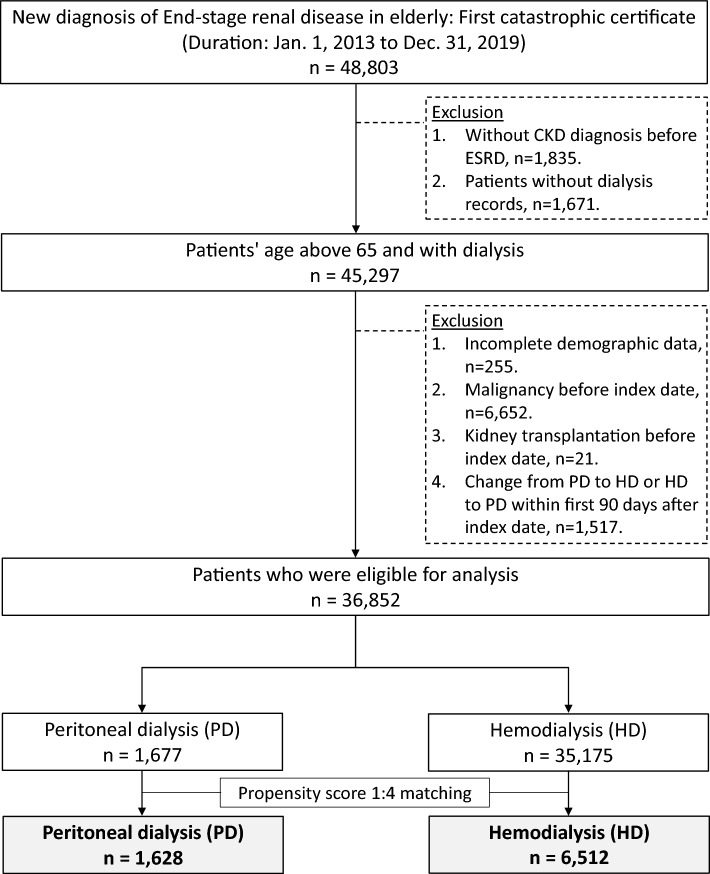
Patient inclusion–exclusion flowchart.

In our study, we utilized data from the NHIRD to evaluate the risks of major adverse cardiac and cerebrovascular events (MACCE), infection, malignancy, or infection and MACCE induced mortality between patients under PD and HD. As shown in Fig. 1, patients aged 65 years or older were included if they obtained ESKD catastrophic illness certification, which indicated new-onset ESKD initiating long-term dialysis, from January 1, 2013, to December 31, 2019. The index date was defined as the date of the next dialysis session after obtaining the catastrophic disease certificate. Exclusion criteria were: 1. Patients without diagnosis of previous chronic kidney disease before index date 2. Patients without records of dialysis 3. Incomplete demographic data 4. Presence of malignancy before the index date 5. History of kidney transplantation before the index date 6. Change from PD to HD or HD to PD within the first 90 days after the index date because numerous prior papers used the 90 days as the dividing point^16–18^. Finally, if a patient undergoes PD on the index date, they would be assigned to the PD group, and vice versa. If patients remained on PD or HD for more than 90 days after the index date and were initially categorized into the PD or HD group, they would continue to be classified in the same group, even if they later switched to the other type of dialysis or underwent kidney transplantation after this 90-day period. However, it's important to note that the observational period for the outcomes we calculated only extends up to the day before they switched to the other type of dialysis or underwent kidney transplantation.

### Covariates and outcomes

In this study, the covariates included age, gender, level of residential urbanization, primary renal diseases, comorbidities, Charlson Comorbidity Index (CCI)^19^, index date, hospitalization history, abdominal surgery history and medication usage. The primary renal diseases in question were hypertensive nephropathy, DM nephropathy, chronic glomerulonephritis (eg, lupus nephritis, IGA nephropathy, and focal segmental glomerulosclerosis), and other forms of renal disease (eg, obstructive nephropathy and interstitial nephritis). Comorbidities were identified if they were reported for more than two outpatient visits or one inpatient stay within the year prior to the index date, which was adopted by numerous previous database studies to define the comorbidities^20,21^. Medications were identified according to the prescriptions within 3 months preceding the index date.

We focused on outcomes such as MACCE (a composite of myocardial infarction, cardiogenic shock, newly diagnosed heart failure, coronary revascularization, malignant arrhythmia, stroke, and cerebrovascular events), infection, malignancy, MACCE-related mortality, infection-related mortality, and overall mortality. MACCE and infection events were identified according to the principal diagnosis during inpatient, outpatient, or emergency room visits. The diagnosis of malignancy occurrence is based on obtaining catastrophic illness certification of malignancy, as the application for this certification requires both evidence from histopathological biopsy and image examinations. All-cause mortality was defined as the patient's name appearing in the Taiwan Death Registry. Due to the potential decline in peritoneal function with prolonged PD duration, a significant number of patients who initially start with PD may switch to HD after approximately 10 years. This disparity in treatment duration between PD and HD could introduce an observational interval imbalance. To mitigate this bias, we have limited our analysis to outcomes within the first 5 years of treatment.

### Statistical analysis

We employed the one-to-four propensity score matching (PSM), in which each patient in the PD group was matched with four counterparts in the HD group. The propensity score was the predicted probability in the PD group derived from logistic regression with all variables listed in Table 1, except for the CCI, which is inherently a composite of other covariates. Then, we can directly compare the outcomes between the two types of dialysis by using the propensity score-matched sample^22–24^. The balance of potential confounders between the groups at the index date was evaluated using the absolute standardized mean difference (ASMD) rather than statistical tests. This is because balance refers to an attribute of the sample and not of the underlying population. An ASMD value of ≤ 0.1 suggested a negligible difference in potential confounders between the groups, whereas an ASMD value between 0.1 and 0.2 indicated a small difference between the groups.

**Table 1:** Baseline characteristics of the patients (peritoneal dialysis group and hemodialysis group).

Variable	Before PSM					After PSM				
	Peritoneal dialysis		Hemodialysis		ASMD	Peritoneal dialysis		Hemodialysis		ASMD
	(*n* = 1677)		(*n* = 35,175)			(*n* = 1628)		(*n* = 6512)		
Age, years (mean, std)	74	7	76	7	0.2939	74	7	74	7	0.0275
Male (n, %)	784	46.75	17,174	48.82	0.0415	767	47.11	2969	45.59	0.0305
Area of residence (n, %)					0.1761					0.0000
Urban	1018	60.70	18,645	53.01		975	59.89	3906	59.98	
Suburban	495	29.52	11,880	33.77		489	30.04	1941	29.81	
Rural	164	9.78	4650	13.22		164	10.07	665	10.21	
Occupation (n, %)					0.2287					0.0000
Dependent	914	54.50	17,234	49.00		894	54.91	3574	54.88	
Civil servant	16	0.95	304	0.86		15	0.92	68	1.04	
Non-manual worker	88	5.25	780	2.22		65	3.99	255	3.92	
Manual worker	382	22.78	10,311	29.31		379	23.28	1518	23.31	
Other	277	16.52	6546	18.61		275	16.89	1097	16.85	
Primary disease for ESKD (n, %)					0.2205					0.1698
Obstructive and interstitial nephritis	25	1.48	670	1.9		22	1.35	123	1.89	
PKD	16	0.95	357	1.01		12	0.74	104	1.6	
Glomerulonephritis	347	20.69	5390	1.01		335	20.58	1069	16.42	
DM nephropathy	808	48.18	20,097	57.13		795	48.83	3380	51.9	
HTN nephropathy	351	20.93	6485	18.44		339	20.82	1338	20.55	
Other	130	7..75	2176	6.19		125	7.68	498	7.65	
Comorbidity (*n*, %)										
Hypertension	1306	77.88	27,996	79.59	0.0419	1272	78.13	5076	77.95	0.0045
Dyslipidemia	500	29.82	11,067	31.46	0.0357	489	30.04	1947	29.9	0.0030
Diabetes mellitus	959	57.19	23,548	66.95	0.2022	945	58.05	3961	60.83	0.0566
Heart failure	230	13.71	6344	18.04	0.1184	228	14	912	14	0.0000
Liver cirrhosis	18	1.07	592	1.68	0.0523	18	1.11	79	1.21	0.0100
Dementia	90	5.37	2662	7.57	0.0896	90	5.53	385	5.91	0.0165
Stroke	173	10.32	4503	12.80	0.0778	172	10.57	670	10.29	0.0090
SLE	6	0.36	85	0.24	0.0212	6	0.37	28	0.43	0.0097
Atrial fibrillation	34	2.03	773	2.20	0.0118	33	2.03	149	2.29	0.0180
Peripheral arterial disease	51	3.04	1150	3.27	0.0131	50	3.07	208	3.19	0.0071
Charlson Comorbidity Index score (mean, std)	3.67	1.59	4	1.69	0.1999	3.7	1.6	3.7	1.6	0.0018
Hospitalization history (n, %)										
MACCE	494	29.46	15,038	42.75	0.2795	491	30.16	1974	30.31	0.0033
Infection	708	42.22	19,611	55.75	0.2733	702	43.12	2795	42.92	0.0040
Abdominal surgery	102	6.08	2304	6.55	0.0192	101	6.20	376	5.77	0.0181
Medication (n, %)										
ACEi/ARB	914	54.5	19,066	54.20	0.0060	887	54.48	3565	54.75	0.0052
Beta-blocker	1048	62.49	22,153	62.98	0.0101	1016	62.41	4119	63.25	0.0175
CCB	923	55.04	20,386	57.96	0.0589	905	55.59	3600	55.28	0.0062
Aspirin/Clopidogrel	558	33.27	14,683	41.74	0.1756	552	33.91	2216	34.03	0.0026
NSAID	852	50.81	20,575	58.49	0.1549	844	51.84	3352	51.47	0.0074
Insulin	651	38.82	17,801	50.61	0.2388	646	39.68	2550	39.16	0.0107
OHA	521	31.07	12,777	36.32	0.1114	515	31.63	2087	32.05	0.0089
Statin	636	37.92	13,239	37.64	0.0059	615	37.78	2488	38.21	0.0089
Donepezil/Rivastigmine	16	0.95	267	0.76	0.0212	16	0.98	59	0.91	0.0079
Follow-up, years (mean, std)	2.17	1.59	2.78	1.96		2.14	1.55	3.07	2.02	

*STD* standardized difference; *PSM* propensity score matching; *ASMD* absolute standardized mean difference; *PKD* polycystic kidney disease; *SLE* systemic lupus erythematosus; *MACCE* major adverse cardiac and cerebrovascular events; *ACEi/ARB* angiotensin-converting enzyme inhibitors/angiotensin receptor blocker; *CCB* calcium channel blocker; *NSAIDs* non-steroidal anti-inflammatory drugs; *OHAs* oral hypoglycemic agents.

*Data were presented as frequency (percentage) or mean ± standard deviation.

^#^Data were presented as percentage or mean ± standard deviation.

The incidence was calculated by dividing the total number of study results during the follow-up period by the person-years at risk. The all-cause mortality between the groups were compared with the Cox proportional hazards model. Other time-to-event outcomes, such as infection-related death and malignancy, were evaluated using a subdistribution hazard model that treated death during the follow-up period as a competing risk. We plotted the Kaplan–Meier curve for all-cause mortality and subdistribution cumulative incidence function for other time-to-event outcomes. In subgroup analysis, we re-estimated PSM to maintain the balance of covariates in each subgroup. A p-value less than 0.05 indicated statistical significance. All statistical analyses were performed using SAS 9.4 (SAS Institute Inc., Cary, NC, USA).

## Results

### Patient characteristics

A total of 36,852 patients, aged more than 65, who were newly diagnosed with ESKD and initiated renal replacement therapy between 2013 and 2019 were identified from NHIRD (Fig. 1). Of these patients, 35,175 received hemodialysis and 1,677 received peritoneal dialysis. Table 1 presents the baseline characteristics of the two groups before long-term dialysis. Before propensity score matching, the HD group had a greater proportion of older patients, a lower rate of dependence, a higher Charlson comorbidity index score, a higher likelihood of hospitalization, a higher prevalence of DM and DM nephropathy and more frequent use of certain medications (i.e., aspirin/clopidogrel, non-steroidal anti-inflammatory drugs, insulin, and oral hypoglycemic agents) compared to the PD group. After propensity score matching, most ASMD values were less than 0.1 and all ASMD values were less than 0.2, indicating that the clinical characteristics between the groups were well balanced. It's worth noting that, whether before or after PSM, the comorbidity of dementia and the history of abdominal surgery between the PD and HD groups showed no significant difference.

### Outcomes

Our objective was to evaluate the five-year outcomes between PD and HD regarding MACCE, infection, malignancy, MACCE-related mortality, infection-related mortality and all-cause mortality in new-onset ESKD patients aged 65 and older. The detailed 5-year outcomes are listed in Table 2.

**Table 2:** Time-to-event outcomes during the 5-year follow-up before and after PSM.

	Peritoneal dialysis		Hemodialysis		PD vs. HD
	Events/Perso*n*-years	Incidence rate* (95%CI)	Events/Perso*n*-years	Incidence rate* (95%CI)	HR (95%CI); p value [Reference group = HD]
Before PSM					
MACCE	323/3287	9.83 (8.75–10.9)	11,924/78,193	15.25 (14.98–15.52)	0.62 (0.56–0.69); < .0001
Infection	888/2677	33.17 (30.99–35.35)	20,179/66,248	31.27 (30.85–31.7)	0.99 (0.92–1.06); 0.7136
PD associated infection**	518/3344	16.20 (14.27–18.13)			
Other infection	514/3055	16.82 (15.37–18.28)	20,179/66,248	31.27 (30.85–31.7)	0.66 (0.6–0.72); < .0001
Malignancy	45/3593	1.25 (0.89–1.62)	2074/94,796	2.19 (2.09–2.28)	0.56 (0.42–0.75); 0.0001
MACCE associated mortality	47/3645	1.29 (0.92–1.66)	2670/97,639	2.73 (2.63–2.84)	0.48 (0.36–0.63); < .0001
Infection associated mortality	203/3645	5.57 (4.80–6.33)	7872/97,639	8.06 (7.88–8.24)	0.67 (0.59–0.78); < .0001
All-cause mortality	504/3645	13.83 (12.62–15.03)	18,430/97,639	18.88 (18.6–19.15)	0.73 (0.66–0.79); < .0001
After PSM					
MACCE	317/3141	10.09 (8.98–11.2)	2124/16,300	13.03 (12.48–13.58)	0.74 (0.66–0.83); < .0001
Infection	868/2546	34.09 (31.82–36.36)	3474/14,391	24.14 (23.34–24.94)	1.28 (1.20–1.37); < .0001
PD associated infection**	507/3201	16.49 (14.42–18.6)			
Other infection	505/2921	17.29 (15.78–18.8)	3474/14,391	24.14 (23.34–24.94)	0.82 (0.75–0.9); < .0001
Malignancy	42/3428	1.23 (0.85–1.6)	413/19,327	2.14 (1.93–2.34)	0.55 (0.40–0.76); 0.0003
MACCE associated mortality	47/3479	1.35 (0.96–1.74)	449/19,970	2.25 (2.04–2.46)	0.62 (0.46–0.84); 0.0019
Infection associated mortality	201/3479	5.78 (4.98–6.58)	1219/19,970	6.1 (5.76–6.45)	0.93 (0.81–1.08); 0.3528
All-cause mortality	498/3479	14.32 (13.06–15.57)	2950/19,970	14.77 (14.24–15.3)	0.98 (0.89–1.07); 0.5868

*Per 100 person-years; *PSM* propensity score matching; *PD* peritoneal dialysis; *HD* hemodialysis; *HR* hazard ratio; *MACCE* major adverse cardiac and cerebrovascular events.

**Hospitalization due to PD-associated peritonitis or PD catheter infection.

**Figure 2:**
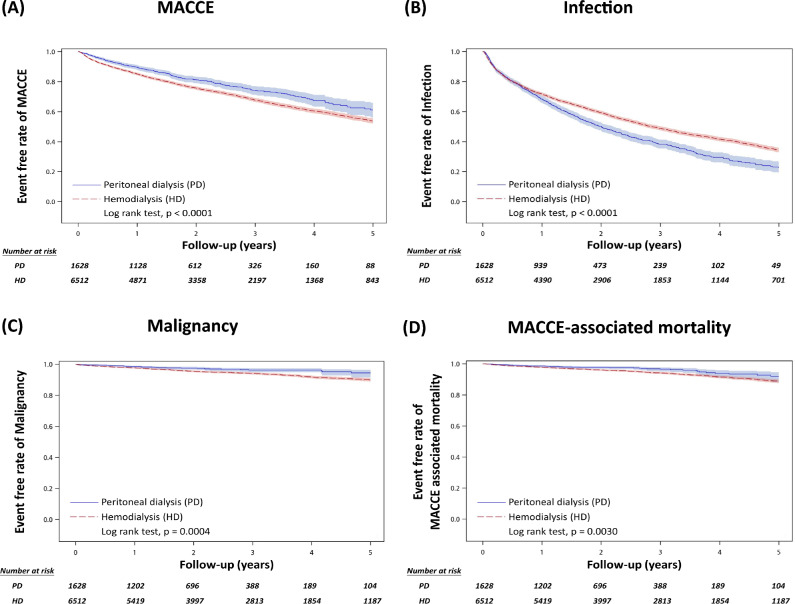
Event free rate for study outcomes after propensity score matching (**A**) MACCE, (**B**) infections, (**C**) malignancy, and (**D**) MACCE-associated mortality. *MACCE* major adverse cardiac and cerebrovascular event.

After PSM, compared to the HD group, the PD group exhibited lower rates (per person-years) of MACCE (10.09% vs. 13.03%, hazard ratio (HR): 0.74, 95% confidence interval (CI): 0.66–0.83), malignancy (1.23% vs. 2.14%, HR: 0.55, 95% CI: 0.40–0.76), and MACCE-associated mortality (1.35% vs. 2.25%, HR: 0.62, 95% CI: 0.46–0.84). On the contrary, the risks of infection were higher in the PD group (34.09% vs. 24.14%, HR: 1.28, 95% CI: 1.20–1.37). However, we found that PD-associated peritonitis or catheter infections represented a large part of infection events in PD group. After excluding PD-associated infections, the risks of other infections were lower in the PD group compared to the HD group. The risks of all-cause mortality (14.32% vs. 14.77%, HR: 0.98, 95% CI: 0.89–1.07) and infection-associated mortality (5.78% vs. 6.10%, HR: 0.93, 95% CI: 0.81–1.08) did not significantly differ between the two groups. Figure 2 presents the cumulative incidence of MACCE, infections, malignancy, and MACCE-associated mortality.

### Subgroup analysis

**Figure 3:**
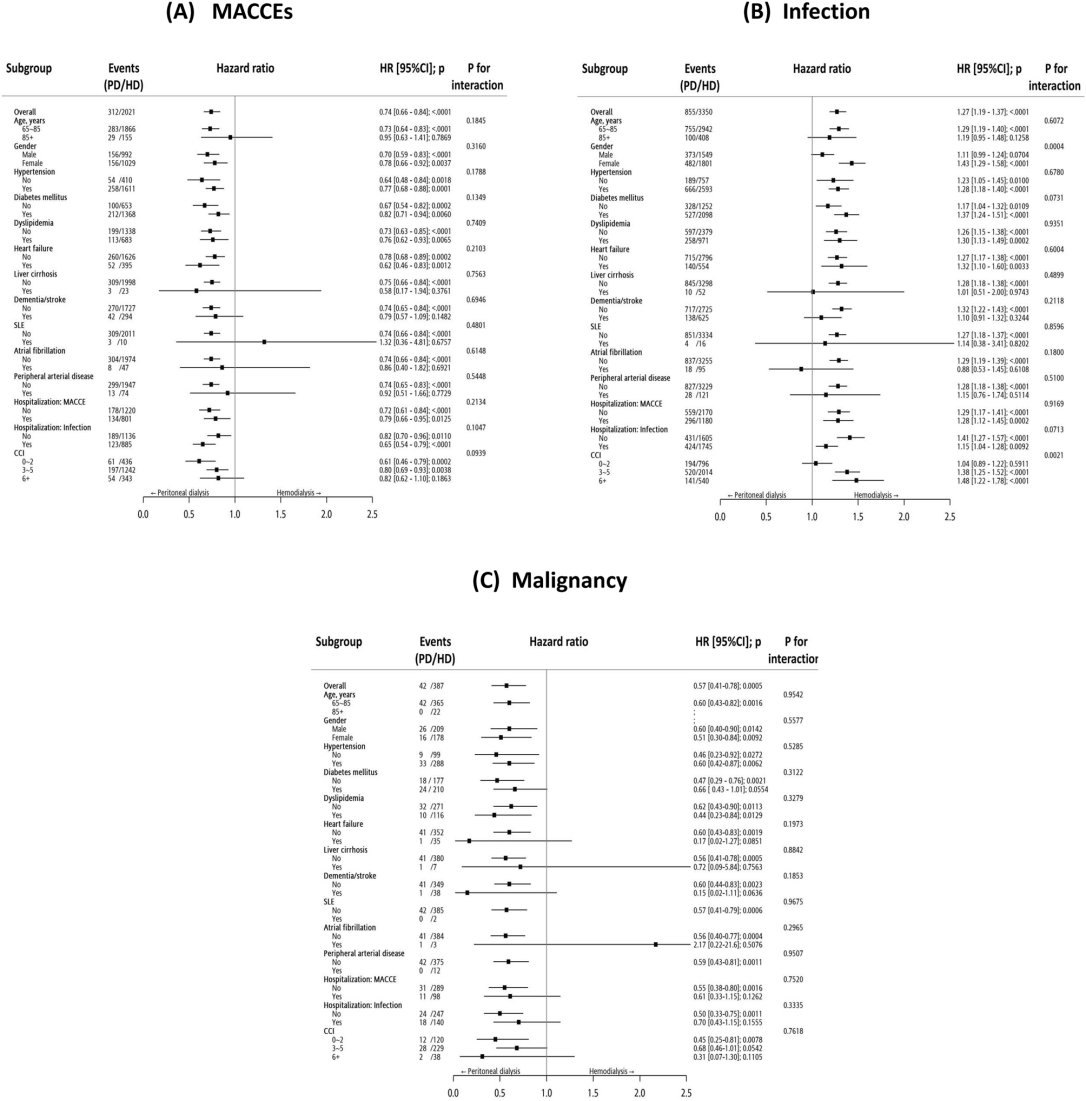
Subgroup analysis of (**A**) MACCE, (**B**) Infection, and (**C**) Malignancy. *HR* hazard ratio. *MACCE* major adverse cardiac and cerebrovascular event.

To ascertain if the benefits of PD or HD are only observed under specific clinical conditions, we further conducted subgroup analyses for MACCE, infection, and malignancy (Fig. 3). Concerning MACCE, PD demonstrated superior performance over HD in most subgroups. About infection, on the contrary, HD outperformed PD in most subgroups, especially in female and a CCI of 3 or higher. In the context of malignancy, PD demonstrated superior performance over HD in most subgroups.

### Mean times of hospitalizations between PD and HD

**Figure 4:**
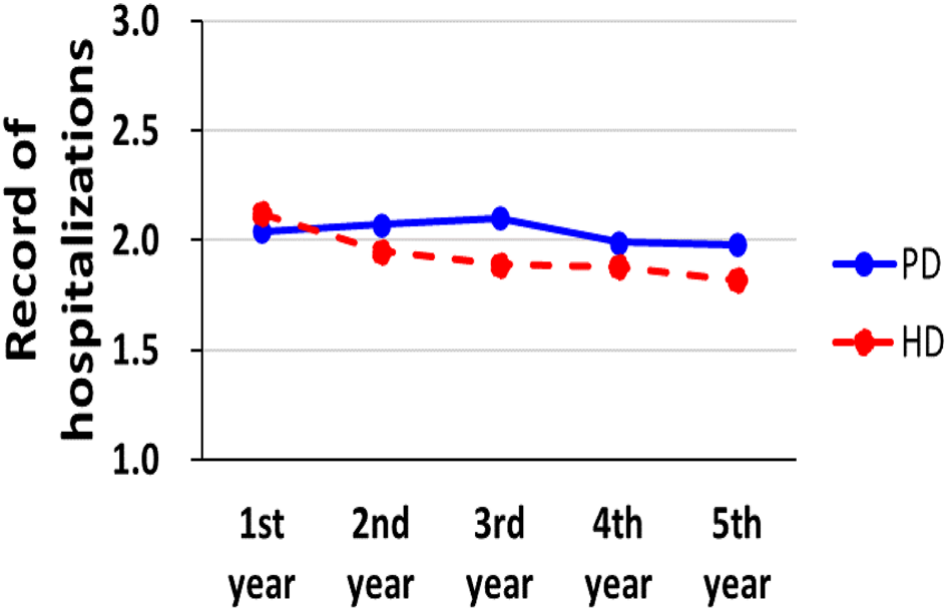
The mean hospitalization times between PD and HD group.

In this study, we observed that patients in the PD group had a lower hospitalization rate during the first year of treatment. However, in the subsequent 2–5 years, they experienced a higher hospitalization rate (Table 3). Despite a statistically significant difference between the two groups, the hospitalization rates were relatively close (Fig. 4).

**Table 3:** The mean hospitalization times between PD and HD.

	Before PSM				After PSM			
	*n*	Peritoneal dialysis	Hemodialysis	*P* value	*n*	Peritoneal dialysis	Hemodialysis	*P* value
Mean times of hospitalizations				0.0018				0.0060
1st year*	22,692	2.03 ± 1.33	2.19 ± 1.55		4508	2.04 ± 1.33	2.12 ± 1.48	
2nd year*	14,543	2.07 ± 1.37	1.99 ± 1.40		3210	2.07 ± 1.37	1.95 ± 1.39	
3rd year*	10,028	2.10 ± 1.55	1.95 ± 1.38		2323	2.10 ± 1.56	1.89 ± 1.33	
4th year*	6889	2.00 ± 1.33	1.94 ± 1.37		1654	1.99 ± 1.30	1.88 ± 1.27	
5th year*	4653	1.96 ± 1.35	1.90 ± 1.29		1127	1.98 ± 1.38	1.82 ± 1.24	

*PSM* propensity score matching.

*After index date.

## Discussion

Because population ageing and the progress of medicine, new-onset ESKD patients aged more than 65 years old increased rapidly in recent decades. Since large portion of elderly ESKD patients would not receive kidney transplantation due to the shorter life expectancy, the decision of receiving PD or HD for this population may be more crucial than younger ESKD patients. By using a large-scale database, this study is aimed to evaluate the risks of all-cause mortality, CV events, infection, and malignancy, which are the most critical health issues in elderly patients, between PD and HD.

Regarding the CV risks, elderly patients under PD seems to be superior to patients under HD. We speculated that the reasons might be multifactorial, including the higher prevalence of myocardial stunning, intradialytic hypotension, cardiac arrhythmias, and transient hypoperfusion of brain during the process of HD^25,26^. In patients under HD, the accumulated uremia toxin and fluid are required to be removed in a short interval and therefore the cardiac hypoperfusion is common. This phenomenon, known as myocardial stunning, can lead to increased CV mortality in ESKD patients. ^27–30^ A prior study from Taiwan have indicated that HD is more likely to result in new onset of coronary artery disease than PD^31^. In addition, the occurrence of arrhythmias in hemodialysis patients is also higher than in peritoneal dialysis patients. A 2019 report on newly diagnosed dialysis in older people found that the chance of new-onset atrial fibrillation was higher in the HD group than the PD group^32^. In addition, intradialytic hypotension is more common in HD patients, which leads to a higher chance of intradialytic arrhythmia in HD patients^33^. Intradialytic hypotension is a cause of morbidity and mortality in elderly patient. ^34^ A 2018 study on hemodialysis in older people pointed out that the process of hemodialysis will lead to a decline in cerebral blood flow. If intradialytic cerebral blood flow declines continue to occur, it may lead to ischemic stroke^35^.

In this study, we observed that HD was superior to PD in infection rates. Infection is the second most common reason for hospitalization among dialysis patients^4^. PD- associated peritonitis represents a significant hurdle for patients considering PD as a dialysis modality. Indeed, in this study, we found PD-associated peritonitis or catheter infection represented a large part of infection events in PD group. A previous study has demonstrated that PD-associated peritonitis is the most prevalent cause for patients transitioning to HD and carries a mortality rate of 2–6%^36^. However, along with the improvement of design of PD dialysis devices and the use of effective intra-peritoneal antibiotics, PD-associated peritonitis has resulted in less mortality and technical failure in recent years^7,37,38^. Thus, although the risks of infection among patients under PD is higher in our study and may consequently result in a slightly higher hospitalization rate, especially since the second year of dialysis, we found no significant difference in all-cause mortality or infection-related mortality between PD and HD. We believed this information is crucial for new-onset ESKD patients to choose dialysis modalities.

The risks of malignancy are another critical issue for elderly patients. Interestingly, this study found that the risks of new-onset malignancy are higher in patients undergone HD compared to PD. The incidence of cancer is higher in patients with ESKD compared to the general population^39–41^. However, there are very few studies investigating which renal replacement modality is associated with the higher incidence of cancer. One prior study indicated that the incidence of cancer was lowest in kidney transplant patients, followed by peritoneal dialysis patients, with the highest incidence observed in hemodialysis patients^42^. For the elderly ESKD patients, our study reached a similar conclusion. A 2017 study in Taiwan informed us that among new dialysis patients, liver cancer is most prevalent, especially in male patients^43^. Other two studies also indicated that age greater than 65 and chronic liver disease are risk factors for cancer in dialysis patients^44,45^. Patients under HD have a higher chance to contact Hepatitis B virus and Hepatitis C virus compared to the patients under PD, thereby may increase their likelihood of developing liver cancer in the future^46,47^. However, to in-depth analyze why the PD patients are associated with lower risks of malignancy and the differences are mainly in which kinds of cancers is beyond the scope of this study. Our research team intends to design further studies to answer these questions comprehensively.

This study has several limitations that should be acknowledged. Firstly, the data analyzed in this study was retrieved from the NHIRD, which lacks certain laboratory data, such as hemoglobin, creatinine, lipid profiles, albumin, and electrolyte levels. This limitation may have affected the comprehensiveness of our analysis. Secondly, being an observational cohort study, it may be susceptible to inherent biases. Nevertheless, conducting randomized clinical trials to evaluate the outcomes of different dialysis modalities would be impractical and ethically challenging. Third, despite the utilization of matching methods, the potential for residual confounding cannot be completely eliminated. For example, if relevant factors (such as dietary habits, smoking, radiation, alcohol, etc.) are not fully adjusted, residual confounding could impact the research findings. Besides, because patients did not randomly select.

PD or HD as long-term treatment, their self-care ability, personality, and familial support system are likely to have influenced their choices, potentially introducing selection bias. Fourth, our study lacked data on cancer-related risk factors, such as smoking and family history of cancer, and information on types of cancer, stages, and mortality rates were not available in our study. These limitations impeded our ability to explore the possible mechanisms behind the increased risk of malignancy occurrence in elderly HD patients compared to those on PD. Our research team plans to design a follow-up study to address this issue.

In conclusion, for the elderly patients with new-onset ESKD, this study found that peritoneal dialysis presents lower risks in MACCE, malignancy, and MACCE associated mortality but is associated with higher risks of infection and slightly higher frequency of hospitalization. The all-cause mortality or infection-related mortality did not differ between PD and HD. This information may help elderly ESKD patients to better choose HD or PD as long-term renal replacement therapy.

## Data Availability

The datasets generated and analysed during the current study are not publicly available because the NHIRD dataset can only be accessed after a qualified investigator submits an application.
